# A Modified Method of Insulin Producing Cells' Generation from Bone Marrow-Derived Mesenchymal Stem Cells

**DOI:** 10.1155/2014/628591

**Published:** 2014-10-22

**Authors:** Paweł Czubak, Agnieszka Bojarska-Junak, Jacek Tabarkiewicz, Lechosław Putowski

**Affiliations:** ^1^Chair and Department of Gynaecology and Gynaecological Endocrinology, Medical University of Lublin, Aleje Racławickie 23 (SPSW), 20-037 Lublin, Poland; ^2^Chair and Department of Clinical Immunology, Medical University of Lublin, W. Chodźki 4a, 20-093 Lublin, Poland; ^3^Centre for Innovative Research in Medical and Natural Sciences, Medical Faculty of University of Rzeszow, 35-959 Rzeszów, Poland

## Abstract

Type 1 diabetes mellitus is a result of autoimmune destruction of pancreatic insulin producing *β*-cells and so far it can be cured only by insulin injection, by pancreas transplantation, or by pancreatic islet cells' transplantation. The methods are, however, imperfect and have a lot of disadvantages. Therefore new solutions are needed. The best one would be the use of differentiated mesenchymal stem cells (MSCs). In the present study, we investigated the potential of the bone marrow-derived MSCs line for *in vitro* differentiation into insulin producing cells (IPSs). We applied an 18-day protocol to differentiate MSCs. Differentiating cells formed cell clusters some of which resembled pancreatic islet-like cells. Using dithizone we confirmed the presence of insulin in the cells. What is more, the expression of proinsulin C-peptide in differentiated IPCs was analyzed by flow cytometry. For the first time, we investigated the influence of growth factors' concentration on IPCs differentiation efficiency. We have found that an increase in the concentration of growth factors up to 60 ng/mL of *β*-FGF/EGF and 30 ng/mL of activin A/*β*-cellulin increases the percentage of IPCs. Further increase of growth factors does not show any increase of the percentage of differentiated cells. Our findings suggest that the presented protocol can be adapted for differentiation of insulin producing cells from stem cells.

## 1. Introduction

Diabetes mellitus is one of major diseases causing heavy burden for many people and countries around the world. These chronic metabolic diseases are caused by absolute (type 1 diabetes) or relative (type 2 diabetes) insulin deficiency leading to hyperglycemia [1, 2]. In both diabetes types, the major determinant for the onset of hyperglycemia and the development of overt disease is an inadequate mass of functional *β*-cells [2]. Currently, there is no perfect cure for diabetes. Insulin injection does not mimic the precise and dynamic regulation of *β*-cells on glucose homeostasis, leading on the long term to the development of complications, for example, renal failure or blindness. It also causes a so-called diabetic foot syndrome and results in a tenfold increase in the risk of limb amputation. Many diabetic patients develop hypertension and cardiovascular diseases [3]. Pancreatic islet cells' transplantation is a promising therapeutic option for diabetes mellitus. However, the lack of pancreas donors remains a major obstacle [1, 4]. In recent years, mesenchymal stem cells (MSCs) derived from various tissues have become a research “hot-spot” because of their potential use in regenerative medicine [1, 4, 5]. MSCs are multipotent nonhematopoietic progenitor cells [6]. MSCs can be cultured in specially defined conditions and their differentiation extends toward the *β*-cell phenotype and the development of insulin producing cells (IPCs) [6]. *β*-cells' replacement is an ideal option for diabetes treatment. Both islet transplantation and islet-like cells could be used in type 1 diabetes, which is caused by autoimmune destruction of pancreatic insulin producing *β*-cells [3, 7]. Unfortunately, in the case of islet transplantation, there is a need for immunosuppression. For that reason, MSCs differentiated into islet-like cells or insulin producing cells (IPCs) could be used [3, 8]. Thanks to that, some serious drawbacks of islet transplantation (like shortage of organ donors or graft rejection) could be overcome [3]. MSCs can be obtained from a patient's autologous tissue, for example, from bone marrow, then expanded and differentiated into IPCs and next transplanted into the same patient [3, 8].

The purpose of the study was to develop a three-stage differentiation protocol for obtaining insulin producing cells from MSCs. Several growth factors, nicotinamide, and high glucose concentration in serum-free DMEM were used as cell differentiation inducers.

## 2. Material and Methods 

### 2.1. MSCs Culture

Human bone marrow stromal cells (MSCs) were purchased from a commercial source (AllCells; catalog number MSC-001F). This cell line was derived from one, nondiabetic Caucasian man. Cells were seeded into 25 cm^2^ culture flasks containing Dulbecco's modified Eagle's medium (DMEM; Biochrom) with 1000 mg/L glucose, 10% fetal bovine serum, 100 U/mL penicillin, and 100 *μ*g/mL streptomycin. MSCs cultures were incubated at 37°C in a humidified atmosphere containing 5% CO_2_. Medium was removed after 48 hours by washing cells with phosphate-buffered saline (PBS; Biochrom) followed by media changes every 2-3 days. After growing nearly to confluence, cells were passaged 3 times by being detached with 0.25% trypsin-EDTA (Gibco) and reseeded at a density of 5 × 10^6^ of cells per 25 cm^2^ culture flask.

### 2.2. Differentiation of MSCs to IPCs

When the entire surface of the culture flask was covered by cells, the process of bone marrow-derived MSCs induction into insulin producing cells (IPSs) was started. We modified the procedure used by Sun et al. [9]. Differentiation of MSCs was assessed in cultures of the third passage using a three-stage protocol.

The first step included cells' incubation (37°C, 5% CO_2_) in serum-free high glucose (4500 mg/L) DMEM with 0.5 mmol/L *β*-mercaptoethanol (Sigma-Aldrich) for 2 days. In the second step, cells were cultured for 8 days in serum-free high glucose DMEM containing 1% nonessential amino acids (Gibco), *β*-fibroblast growth factor (bFGF) (at a concentration of 20 ng/mL, 40 ng/mL, 60 ng/mL, or 100 ng/mL) (Sigma-Aldrich), epidermal growth factor (EGF) (at a concentration of 20 ng/mL, 40 ng/m, 60 ng/mL, or 100 ng/mL) (Sigma-Aldrich), and 2% B27 (Gibco). In the third step, cells were cultured for additional 8 days in serum-free high glucose DMEM containing *β*-cellulin (Sigma-Aldrich) and activin A (Sigma-Aldrich) at a concentration of 10 ng/mL, 20 ng/mL, 30 ng/mL or 50 ng/mL, 2% B27 (Gibco), and 10 mmol/L nicotinamide (Sigma-Aldrich).

### 2.3. Flow Cytometric Analysis

We used a solution of 0.25% trypsin-EDTA (Gibco) for 5 minutes at 37°C to detach the cells. Next cells were suspended in PBS and washed by centrifugation at 1000 rpm for 5 minutes. Then cells were fixed and permeabilized with Cytofix/Cytoperm solution and Perm/Wash buffer (BD Pharmingen), and the nonspecific epitopes were blocked by incubation in normal goat serum (Santa Cruz Biotechnology). Cells were then incubated (20 min at room temperature) with 1 *μ*g monoclonal mouse anti-human insulin antibody (primary antibody recommended for the detection of proinsulin C-peptide of human origin; Santa Cruz Biotechnology). Cells treated with primary antibodies were exposed to secondary antibody solution, goat anti-mouse monoclonal IgG_1_ FITC (Santa Cruz Biotechnology).

**Figure 1:**
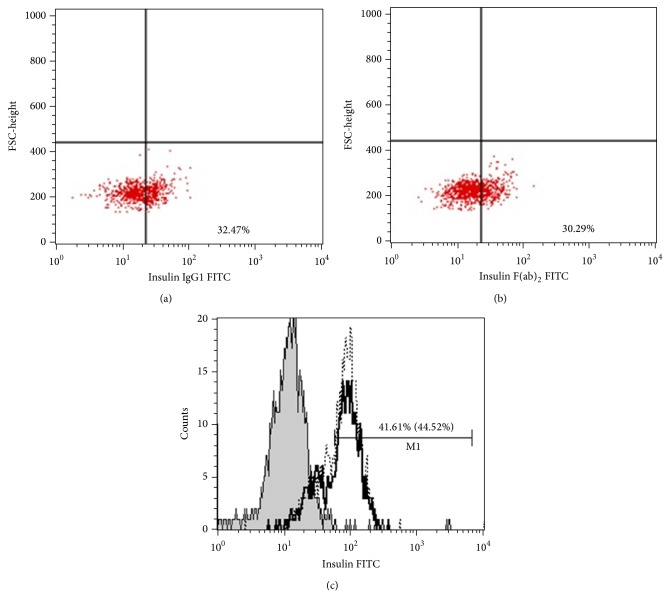
Detection of C-peptide expression by flow cytometry. ((a), (b)) The dot plots show representative data for one sample, illustrating the analysis of C-peptide of proinsulin in MSCs differentiating into insulin producing cells (IPSs). Indirect, intracellular flow cytometry analysis of fixed and permeabilized insulin producing cells (IPSs) stained with mouse anti-human C-peptide of proinsulin primary antibody. Samples treated with primary antibody were next exposed to (a) goat anti-mouse monoclonal IgG_1 _FITC or (b) FITC-conjugated goat anti-mouse IgG F(ab′)_2_. (c) C-peptide expression in differentiated IPCs* from next culture* plotted as a histogram. Grey histogram represents an isotype control. Open histograms represent the expression of C-peptide staining with goat anti-mouse monoclonal IgG_1 _FITC (black solid line) or with FITC-conjugated goat anti-mouse IgG F(ab′)_2_ (dotted line). The numbers in histograms are the percentage of C-peptide positive cells from samples stained with secondary IgG1 or IgG F(ab′)_2_ (between brackets). The *x*-axis corresponds to logarithmic fluorescence intensity and the *y*-axis to relative cell number.

Additionally, in 6 of 16 samples cells treated with primary antibody were exposed to FITC-conjugated goat anti-mouse IgG F(ab′)_2_. The results obtained by those secondary antibodies (isotype specific antibody and IgG F(ab′)_2_) were concordant (Figure 1).

The samples were analyzed by flow cytometry using a Becton Dickinson FACSCalibur instrument. An acquisition gate was established based on FSC and SSC. For each analysis 10,000 events were acquired and analysed using CellQuest Pro software. The percentage of positive cells was measured from a cut-off point set using isotype-matched nonspecific control antibody.

### 2.4. Staining of IPCs by Diphenylthiocarbazone (Dithizone)

Ten mg of diphenylthiocarbazone (Sigma-Aldrich) was dissolved in 10 mL of dimethyl sulfoxide (DMSO, Sigma-Aldrich). Next the stock solution was diluted 1 : 10 in PBS. Cells were incubated in working solution for 30 minutes at 37°C. Finally, cells were examined under a microscope.

### 2.5. Statistical Analysis

We found that distribution of variables did not significantly differ from normal distribution. Quantitative data were expressed as mean ± standard deviation. Mean values between the two groups were compared using the* t*-test for independent samples. We used Statistica 10 PL software for statistical procedures. Differences were considered statistically significant with *P* value ≤ 0.05.

## 3. Results

### 3.1. Insulin Detection by Flow Cytometry

**Figure 2:**
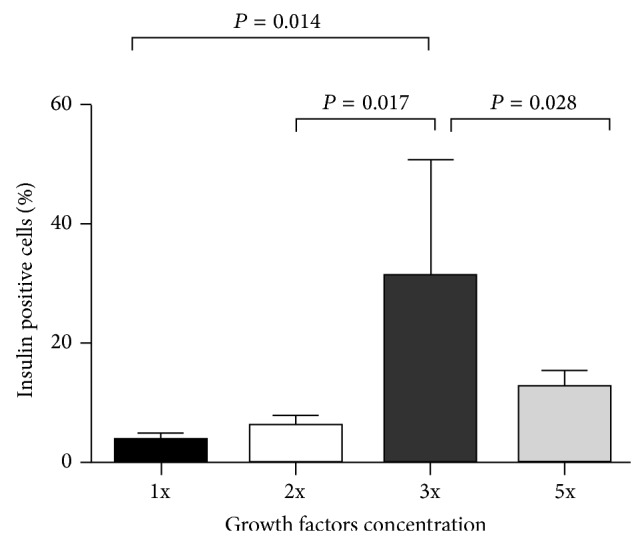
The influence of growth factors' concentration in a growth medium on the number of differentiated IPCs. The largest expression of C-peptide in IPCs was found at concentration of growth factors increased 3 times. An increase in growth factors' concentration above this threshold does not increase the degree of differentiation.

The expression of proinsulin C-peptide from differentiated IPCs was analyzed by flow cytometry (Figures 1(a)–1(c)). We differentiated 16 MSCs cultures into IPCs, using different growth factors concentrations, with respect to the concentration used in the protocol. In three cultures we used *β*-FGF and EGF at a concentration of 20 ng/mL and activin A and *β*-cellulin at a concentration of 10 (1x). In another three cultures we increased the concentration of growth factors twice (2x). In seven cultures the concentration was increased three times (3x) and in three cultures the concentration was increased five times (5x). Flow cytometry analysis of MSCs at 18th day showed that increasing concentrations of growth factors positively affected the ability of MSCs to differentiate into IPCs. At a concentration of 20 ng/mL of *β*-FGF/EGF and 10 ng/mL of activin A and *β*-cellulin the 3.73 ± 1.21% of MSCs differentiated into IPCs positive for C-peptide. That means only about 4% of MSCs responded to such levels of growth factors, differentiated to *β*-like cells and started to produce insulin. At a concentration of growth factors increased to 40 ng/mL for *β*-FGF/EGF and to 20 ng/mL for activin A and *β*-cellulin (2x) 6.59 ± 1.35% IPCs showed C-peptide expression. The highest expression of C-peptide (31.4 ± 19.05%) in IPCs was found at a concentration of growth factor increased three times. Nevertheless, at a concentration of growth factors increased five times the percentage of cells positive for C-peptide significantly decreased (13.00 ± 2.29%) (Figure 2). We found that the positive expression of C-peptide was 1.3 ± 0.5% in negative controls (unstimulated/undifferentiated MSCs).

In flow cytometric analysis two types of secondary antibodies were used (Figures 1(a)–1(c)). The samples treated with primary antibody were next exposed to FITC-conjugated goat anti-mouse IgG F(ab′)_2_ or goat anti-mouse monoclonal IgG_1_ FITC. There was no significant difference between the two staining protocols (*P* = 0.349).

### 3.2. Morphological Changes and Dithizone Staining during the Differentiation

**Figure 3:**
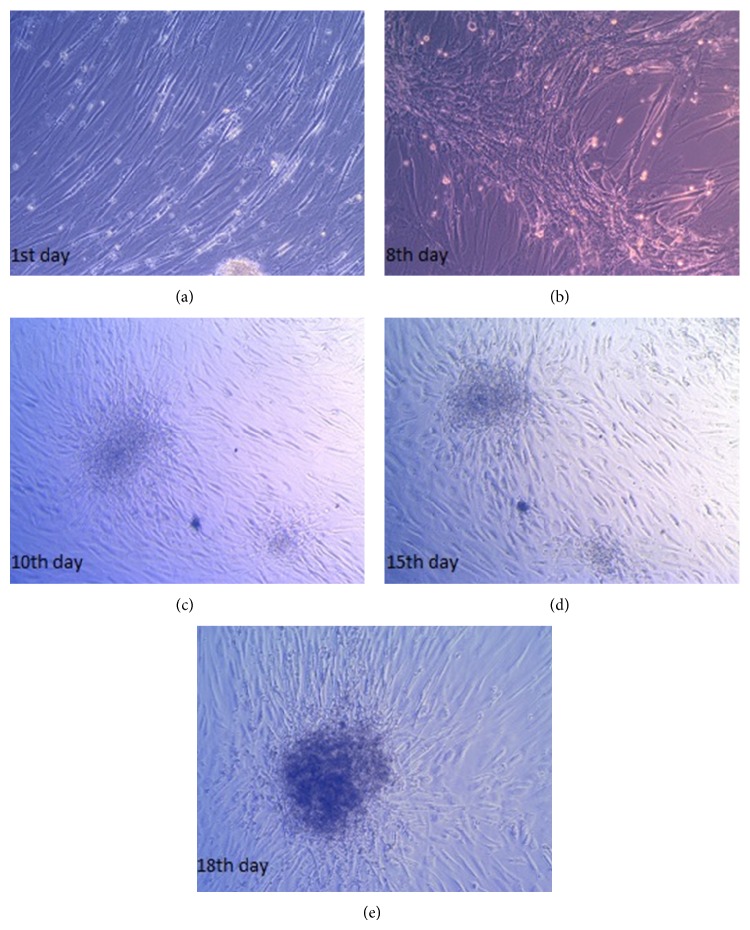
Morphological changes of mesenchymal stem cells during 18 days of differentiation. When observed under an inversed microscope, undifferentiated MSCs looked like long spindle-shaped fibroblastic cells and cultures showed a confluency (1st day). Some cells did not change their morphology during 18 days of differentiation, but many of them migrated (8th day). Cells aggregated in clusters and formed islet-like structures (10th day). Cells created colonies from 10th day and continued to 18th day (×40 magnification).

When observed under an inversed microscope, undifferentiated MSCs looked like long spindle-shaped fibroblastic cells and cultures showed a confluency (1st day; Figure 3). Some cells did not change their morphology during 18 days of differentiation, but many of them migrated (8th day; Figure 3). Cells accumulated together and formed spherical islet-like structures, creating aggregated colonies from 10th day and continued to 18th day (Figure 3).

**Figure 4:**
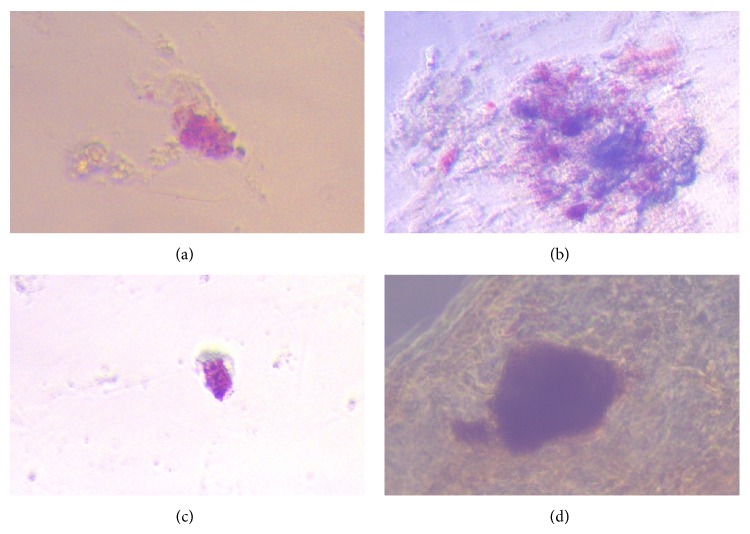
Staining of IPCs by diphenylthiocarbazone (dithizone). The dithizone staining was performed at 18th day. Red staining indicates insulin positivity in aggregated cells (×40 magnification).

To verify the insulin production by the islet-like cells we used diphenylthiocarbazone (dithizone) staining. Dithizone binds to insulin and stains it red. Dithizone staining was performed at 18th day. Red or strong brown staining indicates insulin positivity in aggregated cells (Figure 4). Negative controls showed negative results after dithizone staining.

## 4. Discussion

Type 1 diabetes is characterized by destruction of pancreatic *β*-cells, which is the result of autoimmune response. Both genetic and environmental factors are responsible for the development of the disease. So far, the most commonly used treatment is insulin application, but it has its disadvantages. It seems that the solution for this problem could be pancreas or islet transplantation [10]. Islet transplantation has been shown to be an efficient therapy for type 1 diabetes [11]. However, its clinical usefulness has been limited by immune rejection of transplanted islets and the lack of donor islets cells [2]. It has been suggested that regeneration therapy represents a strategy to overcome the challenges in islet transplantation. MSCs are promoted as an appropriate population in differentiation of insulin producing cells (IPCs) for autologous transplantation [1]. Autologous transplantation prevents autoimmune reactions and can eliminate the need of immunosuppressants' use [3]. IPCs derived from mesenchymal stem cells could be applied as a cell suspension or they could be used with injectable scaffold [5]. MSCs can be isolated from several organs and tissues such as bone marrow, dental pulp, adipose tissues, and umbilical cords [1]. Bone marrow derived MSCs have some significant advantages such as their high number and relatively easy isolation [12]. MSCs are able to differentiate into islet-like cells and possess immunomodulatory abilities, thanks to which it is possible to reduce the risk of immune rejection [10].

IPCs can be obtained by two methods: indirect and direct differentiation. Indirect differentiation uses chemicals (e.g., nicotinamide, growth factors) as inductors. In this method the use of high glucose concentration in the medium is required, since it is a potent inducer of differentiation. The other method, direct differentiation, is based on a modification of genetic material using, for example, viral vectors [4]. Yanai et al. [13] proposed a different approach. They isolated bone marrow MSCs and islets from Lewis rats and used a pulse generator for cell electrofusion. They obtained functional IPCs [13]. It seems that high glucose concentration is a major factor during the process of successful differentiation of MSCs into IPCs. Different research teams used different inducers. Tsai et al. [2] derived IPCs from bone marrow MSCs using serum-free high glucose DMEM with 1% DMSO during the first three-day stage and high glucose DMEM supplemented with 10% FBS during 14 days of the second stage [2]. Tang et al. [14] obtained IPCsusing high concentrations of glucose (17.5 mmol/l) in RPMI 1640 medium supplemented with 10% FCS. By culturing the cells in RPMI 1640 medium containing 1 ng/mL FGF, 10 ng/mL EGF, and 10 ng/mL HGF for 2 months they promoted cellular differentiation. Next, cells were cultured for 7 days in medium containing 5% FCS, 5.5 mmol/l glucose, and 10 mmol/l nicotinamide and 10 nmol/l of exendin 4 [14]. As we can see IPCs can be received using more or less specific inductors of differentiation. The time required for differentiation also varies depending on the reagents used. But according to El-Badri and Ghoneim [3] only 5% of mesenchymal stem cells derived from the bone marrow differentiate into IPCs. In the case of MSC from other sources this percentage is even lower [3].

In the present study, we used indirect differentiation using high glucose concentration (4.5 g/l, 25 mmol/l) among other inducers mentioned above. It is known that glucose promotes cell replication and increases insulin content in the cell [7]. In the current study, differentiating cells formed cell clusters some of which resembled pancreatic islet-like cells. Using dithizone we confirmed the presence of insulin in these cells. What is more, the expression of proinsulin C-peptide in differentiated IPCs was analyzed by flow cytometry. Our data indicate that, in most cases, we obtained more than 5% insulin producing cells mentioned by El-Badri and Ghoneim [3]. In the present study, the highest (mean 31.4 %) number of C-peptide positive cells was found in cultures stimulated with growth factors at a concentration of 60 ng/mL and 30 ng/mL for *β*-FGF/EGF and activin A/*β*-cellulin respectively. We noticed that the increased percentages of differentiated stem cells were associated with higher concentrations of growth factors. We concluded that the concentration of growth factors added to the medium is crucial for the efficiency of IPCs differentiation. On the other hand, we noticed that an increase in the concentration of growth factors above a certain level did not increase the percentage of differentiated cells. So far, our results are based on cytometric analysis and dithizone staining. Our findings suggest that the presented protocol can be adapted for differentiation of insulin producing cells from stem cells. As it is mentioned in the literature, we need about 1 × 10^5^ of islets for proper pancreatic islets transplantation [15]. Each islet contains about 1500 of *β*-cells. This means that during transplantation we need about 1.5 × 10^8^ of *β*-cells. Our cell culture contained about 1 × 10^7^ MSCs, so taking into account the highest achieved yield (approximately 30% of functioning *β*-like cells), there were about 3 × 10^6^ of IPCs. Calculation shows that we need 50 such cell cultures to obtain the desired results, which is not a big number.

Some research teams differentiated MSCs into IPCs, using different methods, but, after clinical application, the results were unsatisfactory. As we can see, only inconsiderable changes that we made during differentiation resulted in significant enhancement of differentiation levels. The results of our investigation show high level of differentiation of MSCs into *β*-like cells, so these findings could be used to clinical applications. Obviously, in the future, good manufacturing practice guidelines should provide guidance for manufacturing, testing, and quality assurance in order to ensure that new IPCs can be safe for human consumption.

Our promising laboratory results could be translated to clinical application in the future. Further studies in diabetic mice with higher quality scores can help find more precise results than* in vitro* studies. At this point, definitely the translation of* in vitro* research findings to useful clinical applications is a major challenge.
